# COVID-19 Vaccine-Induced Latent Autoimmune Diabetes in Adults

**DOI:** 10.7759/cureus.33762

**Published:** 2023-01-14

**Authors:** Klynt Bally, Beisi Ji, Lina Soni

**Affiliations:** 1 Internal Medicine, State University of New York Downstate Medical Center, Brooklyn, USA; 2 Endocrinology, State University of New York Downstate Medical Center, Brooklyn, USA

**Keywords:** pfizer, latent autoimmune diabetes in adults, lada, vaccination, covid-19

## Abstract

Since 2019, COVID-19 has plagued the world with its unfortunate death toll; however, with the introduction of multiple COVID vaccines, mortality and morbidity rates have severely declined. There have been misconceptions surrounding these vaccines, and at the same time, many documented conditions precipitated by the vaccines. This case highlights a speculated relationship between new-onset Latent Autoimmune Diabetes in Adults (LADA) (presenting with diabetic ketoacidosis) and the COVID-19 vaccine. There have been articles suggesting the precipitation of diabetic ketoacidosis /hyperosmolar hyperglycemic syndrome, as well as new-onset diabetes mellitus (DM) with the COVID-19 vaccines but no documented link between LADA and the vaccine. The endpoint of this case is not only to highlight a newfound side-effect of the vaccine but also to urge primary care providers and physicians to closely monitor glucose levels and patient’s A1C after vaccine administration to prevent the formation of these hyperglycemic crises, as well as to consider autoimmune conditions in the differential diagnosis post-vaccination.

## Introduction

Latent autoimmune diabetes in adults (LADA) is a type of slow-onset, immune-mediated insulin deficiency involving progressive destruction of beta-cell function, typically affecting age groups more than 30 years of age. Despite sharing some similarities with type 1 and type 2 diabetes, LADA is a separate entity that should be given equal attention, as patients with this condition are subject to severe complications and hospitalizations if not diagnosed in a timely manner [1]. There have been few cases reported of COVID vaccines precipitating diabetic ketoacidosis (DKA) and hyperosmolar hyperglycemic syndrome (HHS). One case report documented the precipitation of LADA from COVID-19; however, the mechanisms behind this are not fully understood. Furthermore, there have been no reported cases of the COVID-19 vaccines provoking LADA. One hypothesis proposed is that immune dysregulation caused by the vaccine may worsen diabetes and that a similar mechanism underlies the association between COVID-19 infection and diabetes [2]. This would also make sense for the development of LADA since it is an immune-mediated insulin deficiency. What makes this more compelling is that there have been other autoimmune diseases precipitated by COVID vaccines, such as Graves' disease [3]. In this case, we describe a patient who was found to have LADA presenting with DKA after being administered the COVID vaccine.

## Case presentation

A 64-year-old female with hypertension (HTN) and pre-diabetes mellitus (DM) (for 20 years) was brought in by her family for one week of progressive weakness, polyuria, and 24 hours of worsening confusion. Of significance, the patient was administered the second dose of Pfizer-BioNTech COVID-19 vaccine dose one week before her symptoms began. Her baseline mental status was described as alert and oriented to time, place, and person, and she performed her own activities of daily living. Before admission, the patient had an episode of uncomplicated urinary tract infection (UTI) and completed a 10-day course of antibiotics. She had no associated symptoms at the time of presentation, such as chest pain, palpitations, fever, cough, or further urinary symptoms. Otherwise, she had no sick contacts or sources of infection. Her family history is positive for suspected Type 1 DM in her father and Type 2 DM in her mother.

Hospital Course: On arrival, the patient was confused and only oriented to person. Initial vital signs were all stable. BMI was 35.4. Initial laboratory results revealed blood glucose of 1853, pH 7.08, bicarbonate 8, anion gap 39, sodium 120, potassium 8.9, acute kidney injury with creatinine 3.6, all consistent with DKA, and troponins were negative (Table 1).

**Table 1:** Lab values during admission

Tests during admission	Patient’s results	Reference values
Glucose (mg/dL)	1853	70-99
Sodium (mmol/L)	120	136-145
Potassium (mmol/L)	8.9	3.5-5.1
Bicarbonate (mmol/L)	8	21-31
Anion gap (mmol/L)	39	10-20
Creatinine (mg/dL)	3.6	0.6-1.2
Beta-hydroxybutyrate (mmol/L)	>8	0.02-0.27
pH	7.08	7.31-7.41
Troponin (ng/mL)	0.02	<=0.15
Hemoglobin A1C (%)	15.1	4.1-5.7
Glutamic acid decarboxylase antibody (lU/mL)	>250	<5
C-peptide (ng/mL)	0.19	0.8-3.85
Islet cell antibody	Negative	Negative

Urinalysis was negative for a UTI, and urine culture was negative. The patient was eventually admitted to the medical ICU (MICU) for management of her DKA, given intravenous fluids and insulin drip, and showed marked clinical and laboratory improvement. She was ultimately downgraded to medical floors after two days and was transitioned to 25 units Lantus (Sanofi, Bridgewater, USA) and 9 units aspart. Endocrinology was consulted and followed the patient in the hospital. Other labs done during admission revealed a hemoglobin A1C of 15.1% and increased glutamic acid decarboxylase antibodies (GAD) to > 250 with corresponding low C peptide levels suggesting Type 1 DM. Beta-hydroxybutyrate was >8. Baseline A1C was ~6.0. The patient was followed up two months after discharge and was compliant with her insulin regimen of Novolog (Novo Nordisk, Bagsvaerd, Denmark) 9 units three times daily and Lantus 27 units nightly, achieving excellent glycemic control, and her doses were tapered accordingly. Follow-up labs post-admission revealed persistent elevated GAD levels, and C peptide remained low, further confirming LADA (Table 2).

**Table 2:** Lab values after admission

Tests at three months clinic follow up	Patient’s results	Reference values
Glutamic acid decarboxylase antibody (lU/mL)	>250	<5
C-peptide (ng/mL)	0.69	0.8-3.85
Islet cell antibody	Negative	Negative

## Discussion

With the evolution of the COVID-19 pandemic since 2020, there has been a shift in mortality trend since the introduction of COVID vaccinations. According to CDC statistics, as of February 2022, in the US alone, the average daily new cases of COVID-19 decreased by 37.7%, with hospitalizations decreasing by 29.9% and deaths dropping by 18.8%. Corresponding to these values, 76.3% of the US population received at least one dose of the vaccine, and 64.8% were fully vaccinated. There is clear evidence that vaccines work; however, little is known about their side effects. Many articles and case reports have since been published showcasing possible side effects. It is already known that COVID-19 can precipitate or worsen diabetes. A meta‐analysis of eight studies of nearly 3,700 patients revealed an average of 14.4% for new-onset diabetes on admission in patients hospitalized with COVID-19 [4]. Patients with new-onset diabetes on admission for COVID-19 were found to significantly increase in mortality compared with patients with pre-diabetes or preexisting diabetes [4].

Regarding the vaccine and diabetes, few cases have been reported with new-onset DM with DKA/HHS after administration of the vaccine, with one case series describing hyperglycemic crises precipitated by Pfizer and Moderna vaccines. Our case illustrates a link between COVID-19 vaccination, specifically Pfizer-BioNTech, with latent autoimmune diabetes in adults (LADA). Several hypotheses, which were compiled in a 2021 article [2], have been proposed to explain how SARS-CoV-2 may trigger new-onset or exacerbate known DM, including: (1) SARS-Cov can infect pancreatic beta cells directly, or cause direct or bystander destruction of pancreatic beta cells by binding to pancreatic angiotensin-converting enzyme 2 (ACE2) receptors [2,4,5]; (2) Releasing of cortisol and catecholamines because of the acute stress caused by SARS-CoV-2; (3) disruption of the renin-angiotensin-aldosterone and angiotensin 1-7 balance, favoring upregulation of the pro-inflammatory angiotensin II pathway with downregulation of the vasodilatory, protective angiotensin 1-7 pathway with local, detrimental effects at the pancreas [5, 6]; or (4) Infection will lead to dysregulation of the immune system, triggered systemic inflammatory response and release of cytokines, a “cytokine storm” [7, 8].

Another theory was suggested in an article showcasing a link between COVID-19 vaccine and Graves' disease cases [3] - they state the vaccine contains four lipids, of which two are polyethylene glycol (PEG) lipid conjugates that stabilize the lipid nanoparticles and reduce the activity of non-specific binding proteins (22). PEGs may act as an adjuvant and induce an immune response in predisposed individuals, as there are reports of reactions to PEGs (23). Since Graves' disease and LADA are autoimmune pathologies, this theory possibly explains both. This case can further exhibit not only the link between COVID-19 vaccines and diabetes but also the link with autoimmune conditions entirely. This obese patient, who has been pre-diabetic for more than 20 years, suddenly presented with DKA only a few days after COVID-19 vaccination, with positive autoantibodies and requiring insulin. In light of her completing a course of antibiotics for her uncomplicated UTI and the admitting urinalysis was negative for any infection, in addition to a negative urine culture - it is unlikely that this was due to the UTI.

## Conclusions

All COVID-19 vaccines have proven to be effective in the fight against the SARS-CoV-2 Virus and the pandemic, and have all been deemed to be relatively safe. At the time of this case report, there have been no other documented cases speculating the link between the vaccine and LADA. Not only does this case further reiterate the point that prompt screening for DM in susceptible patients following administration of COVID-19 vaccine should be considered by the treating providers, it further stresses the point that we need to take into consideration autoimmune diseases as a cause of patient’s symptoms post vaccination.
